# Assessment of real-world evidence research competencies in federated research networks: a maternal health fellowship program evaluation

**DOI:** 10.1093/jamiaopen/ooag128

**Published:** 2026-07-14

**Authors:** Haeun Lee, Benjamin Martin, Andreea A Creanga, Evan Minty, Asieh Golozar, Cindy X Cai, Cynthia Sung, Sean O’Rilley, Khyzer Aziz, Paul Nagy

**Affiliations:** Biomedical Informatics and Data Science, Johns Hopkins School of Medicine, Johns Hopkins University, Baltimore, MD, 21205, United States; Biomedical Informatics and Data Science, Johns Hopkins School of Medicine, Johns Hopkins University, Baltimore, MD, 21205, United States; Department of Gynecology and Obstetrics, The Johns Hopkins University School of Medicine, Baltimore, MD, 21287, United States; Faculty of Medicine, O’Brien Institute for Public Health, University of Calgary, Calgary, Alberta, 2500, Canada; Biomedical Informatics and Data Science, Johns Hopkins School of Medicine, Johns Hopkins University, Baltimore, MD, 21205, United States; Nemesis Health, New York, NY, 10009, United States; Biomedical Informatics and Data Science, Johns Hopkins School of Medicine, Johns Hopkins University, Baltimore, MD, 21205, United States; Wilmer Eye Institute, Johns Hopkins School of Medicine, Johns Hopkins University, Baltimore, MD, United States; Center of Regulatory Excellence, Duke-NUS Medical School, Singapore; Biomedical Informatics and Data Science, Johns Hopkins School of Medicine, Johns Hopkins University, Baltimore, MD, 21205, United States; Biomedical Informatics and Data Science, Johns Hopkins School of Medicine, Johns Hopkins University, Baltimore, MD, 21205, United States; Department of Neonatology, The Johns Hopkins University School of Medicine, Baltimore, MD, United States; Biomedical Informatics and Data Science, Johns Hopkins School of Medicine, Johns Hopkins University, Baltimore, MD, 21205, United States

**Keywords:** real-world evidence, real-world data, observational research, core competency, professional development

## Abstract

**Background and Significance:**

Generating real-world evidence (RWE) approaches across federated evidence networks based on real-world data requires learning new methodological skills. However, standardized frameworks for defining and assessing competencies for large-scale RWE research remain limited. This study aims to develop an assessment framework for RWE studies and evaluate the competencies of RWE research professionals.

**Methods:**

We adapted the Joint Task Force (JTF) Core Competency Framework for RWE research using the Observational Medical Outcomes Partnership (OMOP) Common Data Model. Through iterative expert review, 4 new domains were created, including Ethics and Governance, Protocol Development, Study Operations, and Data Analysis and Informatics, and 4 original JTF domains were adapted. Participants completed pre- and post-training self-assessments on a 10-point scale. Paired *t*-tests with effect sizes were used to evaluate changes in competency scores.

**Results:**

Seventeen participants from academic, data, and clinical roles completed the assessment. Mean self-reported competency scores increased by 2.5 points across the 8 competency domains, with statistically significant changes observed in all domains. The largest gain was observed in Leadership and Professionalism (mean increase 3.66; 95% CI, 2.69-4.62).

**Discussion:**

The RWE research competency assessment tool captured changes in self-reported skill proficiency, perceived readiness to conduct RWE studies, and methodological knowledge gaps. These findings suggest the potential to identify competency gaps, tailor educational interventions, and inform quality benchmarks for RWE research training programs.

**Conclusion:**

This study developed and evaluated a competency assessment framework specifically designed for RWE research using standardized healthcare data, providing a tool for training program evaluation and workforce development support.

## Background and significance

Training in Epidemiology and Pharmacoepidemiology has long provided the methodological foundation for observational clinical research and for generating real-world evidence (RWE).^1^ These disciplines have offered structured instruction in study design, causal inference, evaluation of treatment effectiveness and safety, patterns of medication use, and surveillance for adverse events, equipping researchers with core competencies required for rigorous RWE studies.^2^^,^^3^ However, such training has largely focused on analyses using centrally curated datasets, such as prospective cohorts or retrospective chart reviews, and has not leveraged the scale, complexity, and heterogeneity of analyses based on large, routinely collected digital health data.^4–6^ Real-world data (RWD) offer opportunities to generate novel and potentially more generalizable evidence, yet specialized training is necessary to use these data meaningfully at scale. To this end, researchers must be trained to work within distributed, multi-institutional data environments, including the use of standardized data models, handling cross-site data heterogeneity, and reproducible analyses.

The use of RWD to generate RWE has expanded across biomedical research, healthcare systems, and policy-making.^4^^,^^7^^,^^8^ Regulatory agencies, including the U.S. Food and Drug Administration (FDA), have released guidance and evaluation frameworks to assess the appropriateness of data sources, analytic methods, and study designs for RWE generation. The National Center for Advancing Translational Sciences (NCATS) has established the Health artificial intelligence (AI) and RWD Training Hub to provide open-access educational resources for learners at all levels, from clinicians and researchers to data scientists and policymakers, supporting the biomedical community in building expertise and capacity in health AI, and RWD analysis.^9–11^ Moreover, the Observational Health Data Sciences and Informatics (OHDSI) open science community offers various training opportunities and modules available through the European Health Data & Evidence Network (EHDEN) Academy and *The Book of OHDSI* to help new researchers learn the methods and tools needed to increase reproducibility and transparency of RWE studies.^12^^,^^13^ These initiatives illustrate the increasing emphasis on developing training programs to support the RWE research workforce.

Despite advances in data infrastructure and training resources, systematic training infrastructure and structured competency evaluation for conducting large-scale RWE research remain underdeveloped. In particular, existing RWE guidelines describe methodological components of RWE studies but do not explain how it is translated into implementation within data-driven RWE studies using standardized observational healthcare data.^14–16^ While established competency frameworks such as the Joint Task Force (JTF) model exist, they were designed for clinical trial research and do not address the distinct methodological and operational requirements essential for RWE research.^17–19^ This gap makes it challenging to evaluate researchers’ competence in conducting RWE studies, limiting the ability to establish standardized competency criteria for workforce development.

Large-scale RWE studies require competencies that differ from those used in traditional clinical trial research due to their non-interventional design and heterogeneous data. First, the data sources differ. RWE studies draw from diverse RWD such as electronic health records (EHRs), administrative claims, and registries, which were originally collected for clinical care or operational purposes rather than research.^20^ Study execution therefore requires an understanding of data integration and interoperability, as well as expertise in data curation such as data cleaning, harmonization, and standardization to address missingness, inconsistency, and variation in data quality.^21^ Second, while these studies offer higher external validity through larger, more representative populations under real-world conditions, they are susceptible to confounding factors.^22^^,^^23^ Addressing these limitations requires the selection of appropriate RWE study designs across multiple data sources and robust statistical methods such as propensity scoring and negative controls to mitigate bias and confounding in RWE analyses.^24^^,^^25^ Third, federated RWE research requires a multidisciplinary skill set encompassing data harmonization, advanced data analytics to interrogate complex RWD, the use of common data standards for interoperability, and coordination across diverse stakeholders.^26–28^ However, current competency instruments such as the Clinical Investigator Competency & Readiness Program (CICRP) and JTF for Clinical Trial Competency surveys focus on traditional clinical trial domains such as biostatistics, the medicine development process, and regulatory compliance, providing limited guidance for researchers working with standardized healthcare data or federated analytic networks.^18^^,^^29^ To address this gap, our team developed competency items specific to RWE studies within federated healthcare data networks and assessed participants’ capabilities before and after training to identify areas for continued workforce development.

## Methods

### Instrument development

We adapted the JTF Leveled Core Competency Framework for the Clinical Research Professional (Version 3.1) to develop an assessment instrument tailored to RWE research. Following Hinkin’s framework for scale development, we reviewed and refined the JTF competency domains to align with the technical and methodological demands of large-scale RWE studies.^30^ We then performed expert content validation to confirm that the updated domains were appropriate and representative of the competencies we aimed to assess.

To develop competency areas specific to RWE research, we conducted a literature review of existing quality improvement instruments, including the CICRP and the JTF Core Competency Survey, and reviewed the OHDSI research protocol and related literature on RWE generation.^31^^,^^32^ We identified key knowledge and skills required for RWE research competency: generating evidence from RWD, leveraging standardized data models for reproducible research, conducting effective network studies, designing robust RWE research protocols, mastering OHDSI analytical tools, producing scientific publications and grant proposals, and building collaborative relationships within research networks. Based on these identified elements and our program curriculum (Appendix 3), we developed competency areas for each domain and mapped these areas onto three core dimensions: (1) career development, (2) research practice, and (3) professional networking (Appendix 1). This process yielded 4 new domains addressing RWE research-specific competencies: RWE research ethics and data governance, RWE research protocol development, RWE study operations, and data analysis and informatics (Table 1). The other 4 original JTF domains (scientific concepts and research design, study and site management, leadership and professionalism, and communication and teamwork) were retained but adapted to reflect RWE research requirements. In addition to competency self-assessment items, the survey collected demographic information, including the highest degree obtained prior to fellowship entry, current professional role, and years of experience in that role. Each competency item was rated on a 10-point scale ranging from 0 (no knowledge) to 10 (advanced/expert level).

**Table 1.** Mapping of JTF clinical research competencies to RWE research competency domains.

JTF domain	JTF key elements	RWE research domain	RWE research key elements
1. Scientific concepts and research design	• Clinical research hypotheses from literature	1. Scientific concepts and research design	• Research questions suitable for RWE studies
	• Statistical, epidemiological, operational study design		• Real-world data availability assessment
	• Therapeutic and comparative effectiveness		• Target/comparator/outcome cohorts using standardized tools
			• Real-world effectiveness and population health
2. Ethical and participant safety considerations	• Human subject protection (national/international)	2. RWE research ethics and governance	• Multi-institutional EHR databases protection
	• Privacy regulations across phases		• Data governance across institutions
	• Vulnerable populations and safeguards		• Regulatory compliance for RWE research
3. Investigational products development and regulation	• Medicine development process	3. RWE research protocol development	• Clear research questions for cross-institutional work
	• Roles of institutions in drug development		• Computable phenotypes using standardized data
	• Regulatory pathways		• Analysis plans with appropriate statistical methods
			• Protocol components for RWE studies
4. Clinical study operations (good clinical practice)	• Clinical trials within development plan	4. RWE study operations	• Multi-institutional studies within OHDSI framework
	• Clinical investigation team roles (GCP)		• RWE research team roles (OHDSI guidelines)
	• Monitoring processes		• Collaborative platforms and version control
5. Study and site management	• Financial, timeline, personnel management	5. Study and site management	• Timeline and personnel for multi-institutional studies
	• Risk management and quality improvement		• Risk management and quality in RWE research
	• Patient recruitment and progress tracking		• Progress tracking in collaborative networks
6. Data management and informatics	• Biostatistics and informatics role	6. Data analysis and informatics	• Data transformation, CDM harmonization, and understanding of data quality
	• Data flow in clinical trials		• Open-source tools for data harmonization
	• Electronic data capture (EDC)		• Statistical methods for large-scale analyses
	• Data quality assurance and SOPs		• Cohort generation and standardized analytical frameworks
			• Reproducibility across multi-institutional networks
7. Leadership and professionalism	• Leadership, management, mentorship principles	7. Leadership and professionalism	• Leadership for RWE research teams
	• Professional guidelines and ethics codes		• Ethics in collaborative networks
8. Communications and teamwork	• Sponsor-CRO-site communication	8. Communication and teamwork	• Study leads, data partners, OHDSI collaborators
	• Communication to colleagues and public		• Communication to clinicians and public
			• RWE research findings dissemination

Abbreviations: CDM, Common Data Model; CRO, Contract Research Organization; EHR, Electronic Health Record; GCP, Good Clinical Practice; JTF, Joint Task Force for Clinical Trial Competency; OHDSI, Observational Health Data Sciences and Informatics; OMOP, Observational Medical Outcomes Partnerships; RWD, Real-World Data; RWE, Real-World Evidence; SOP, Standard Operating Procedures.

To further refine the instrument, we conducted iterative reviews with a multidisciplinary panel of experts in healthcare and biomedical informatics. These reviews evaluated the clarity, relevance, and comprehensiveness of each item and ensured that the instrument captured meaningful RWE research competencies. Both original and newly developed items were examined for conceptual equivalence and content validity. We also followed the American Association for Public Opinion Research (AAPOR) Best Practices for Survey Research to ensure the quality and reliability of the survey findings.^33^ The finalized survey instrument is provided in Appendix 2.

### Training program

The Maternal Health Data Science Fellowship was an 8-month program (September 2024-April 2025) designed for early-stage researchers in academic medicine with a focus on maternal health, combining education, networking, and practice. Training was delivered through monthly synchronous sessions and self-paced online modules. Fellows participated in two monthly sessions, a Maternal Health Fellowship call and a Pregnancy and Reproduction Epidemiology Working Group (PHReG) session, along with asynchronous coursework through the EHDEN Academy and assigned reading from The Book of OHDSI and related publications. The curriculum covered OHDSI and observational research fundamentals, research question development and feasibility, data quality, phenotype development, study design, and key methodological approaches, focusing on standardized data models (Observational Medical Outcomes Partnership Common Data Model [OMOP CDM]) and multi-institutional RWE studies within a federated network. Additional topics included analytic tools, data characterization, clinical terminologies, cohort definition, and study protocol development. Participants developed project proposals and were organized into small teams to support collaborative study development. Further details of the curriculum are provided in Appendix 3.

### Participant recruitment and assessment

Participants were recruited from the OHDSI Maternal Health Fellowship Program, a training initiative supported through the NIH Implementing a Maternal Health and Pregnancy Outcomes Vision for Everyone (IMPROVE) initiative. Fellows were selected through an open application process based on CV and research proposal review. During the program, engagement was tracked through attendance at synchronous sessions, and training completion was assessed based on session attendance, completion of EHDEN Academy modules, and a final project presentation. At the end of the program, survey invitations were sent via email to all 25 fellows between August 11 and September 11, 2025, and responses were collected through Google Forms. Competency was assessed using a retrospective pre-post design, in which participants rated their perceived competency before and after training at the same time point.

### Data analysis

Survey responses were aggregated using Google Spreadsheets. We compared pre-training and post-training competency scores by calculating the mean and standard deviation (SD) for each time point. To evaluate changes in competency following the fellowship program, paired *t*-tests were used to assess mean differences, and 95% confidence intervals (CI) were reported for the mean difference. Effect sizes were estimated using Cohen’s *d* to supplement statistical significance. All analyses were performed in RStudio (version 4.4.2).

## Results

### Sample characterization

**Figure 1.**
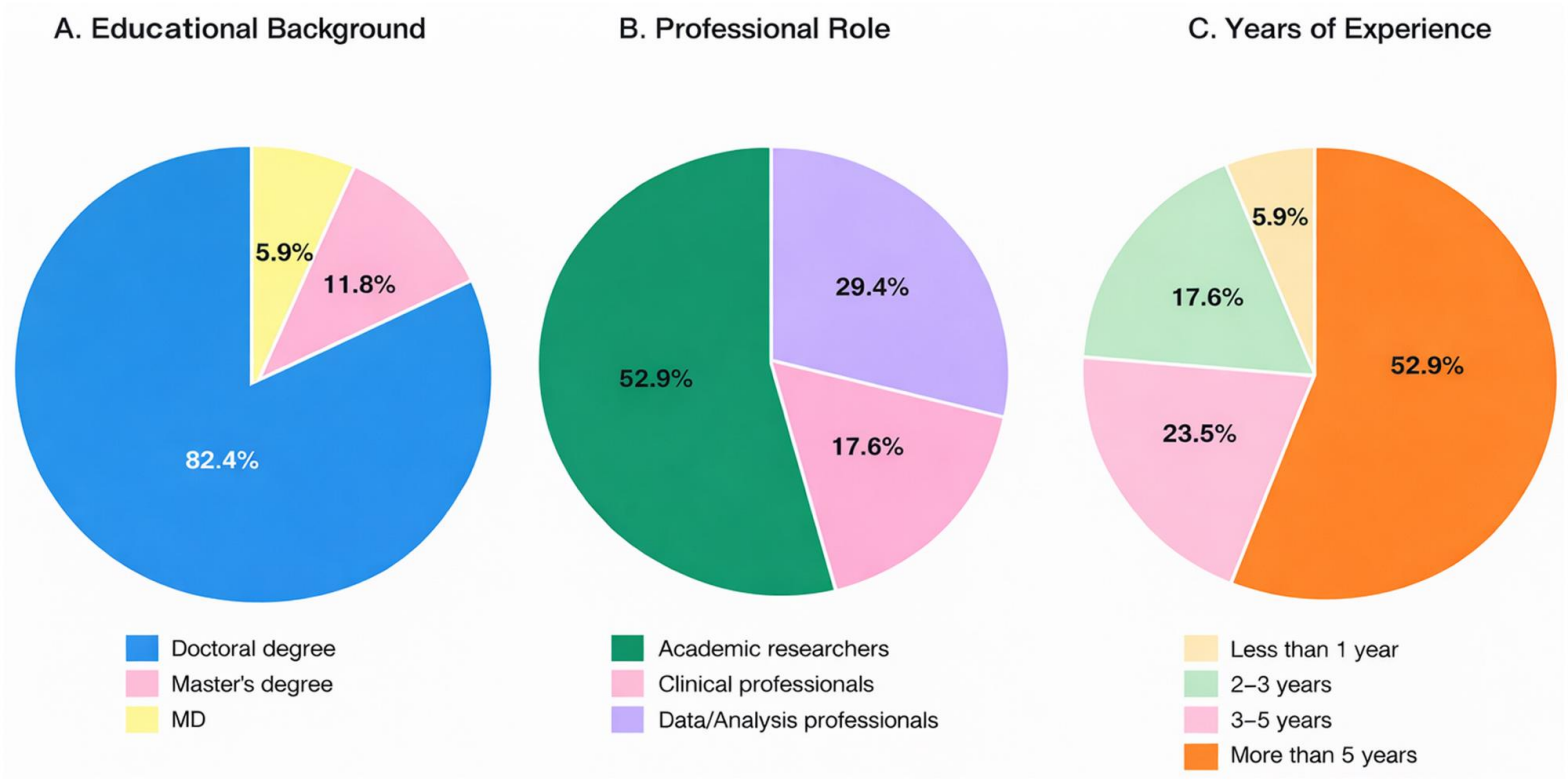
Characteristics of study participants in the assessment. Pie chart showing the distribution of study participants according to (A) educational background (B) professional role, and (C) years of professional experience.

Of the 25 invited participants, 17 completed the questionnaire (68% response rate). Educational backgrounds varied, with the majority holding doctoral degrees (77.8%). Half of the respondents had more than 5 years of clinical research experience (50%), followed by 3-5 years (22.2%) and 2-3 years (16.7%). Nearly half of the sample identified as investigators or academic research faculty (47.1%), including associate clinical professors, nurse scientists, postdoctoral scholars, research scientists, PhD candidates, and assistant professors (Figure 1). Among respondents, 29.4% were data and analytics professionals, including biostatisticians, data scientists, informaticians, and epidemiologists. Clinical professionals, including practicing clinicians, represented the smallest group at 17.6%.

#### Knowledge and competencies in all domains

Table 2 presents self-reported competency scores across 8 domains before and after the fellowship program. All domains showed statistically significant changes following training (all *P* < .001). The overall average score increased from 1.92 to 3.66, indicating a general increase in perceived competency following training. The largest gains were observed in Domain 7: Leadership and Professionalism (mean change = 3.6 points, 95% CI, 2.69-4.62, *d* = 2.03), followed by Domain 6: Informatics and Data Analysis (mean change = 3.38, 95% CI, 2.49-4.25, *d* = 2.04). Domain 1: Scientific Concepts and Research Design showed a mean change of 3.35 (95% CI, 2.38-4.32, *d* = 1.78), consistent with increased self-reported foundational research literacy. In addition, moderate-to-large effects were present in RWE governance, research protocol development, operational execution, study management, and communication/teamwork (*d* = 1.13-1.49).

**Table 2.** Average self-assessed competency scores before and after the program by domain.

Domain	*N*	Pre-training score mean (SD)	Post-training score mean (SD)	Change (95% CI)	*P* value	Cohen’s *d*
1. Scientific concepts and research design	17	3.25 ± 2.10	6.61 ± 1.88	3.35 (2.38, 4.32)	<.001***	1.78
2. RWE research ethnics and governance	17	5.88 ± 1.98	7.84 ± 1.32	1.96 (1.27, 2.65)	<.001***	1.45
3. RWE research protocol development	17	5.96 ± 2.44	7.88 ± 1.30	1.92 (1.05, 2.79)	<.001***	1.13
4. RWE study operations	17	2.82 ± 2.00	5.04 ± 1.94	2.22 (1.45, 2.98)	<.001***	1.49
5. Study and site management	17	2.96 ± 2.43	5.22 ± 2.34	2.25 (1.43, 3.08)	<.001***	1.4
6. Informatics and data analysis	16	2.41 ± 1.49	5.79 ± 1.76	3.38 (2.49, 4.26)	<.001***	2.04
7. Leadership and professionalism	16	2.09 ± 1.53	5.75 ± 1.95	3.66 (2.69, 4.62)	<.001***	2.03
8. Communications and teamwork	16	4.56 ± 2.15	6.97 ± 1.49	2.41 (1.43, 3.38)	<.001***	1.32

Abbreviations: *N*, Number of Respondents; RWE, Real-World Evidence.

Scores based on 0-10 scale where 0 = little knowledge and 10 = high proficiency; paired *t*-tests used; Cohen’s *d* shows effect size.

*** *P* < .001.

### Competency area outcomes within each domain

Within the foundational knowledge and design domains (Scientific Concepts & Research Design, Ethics & Data Governance, and Protocol Development), the largest increase was observed in understanding OHDSI study designs for cross-institutional RWE research, with a mean change of 3.94 points (Table 3). Participants also showed increases in understanding data governance frameworks and regulatory requirements for multi-institutional RWE research using EHRs, with a mean change of 2.53 points. Among protocol development competencies, defining computable phenotypes for RWE research using standardized healthcare data increased by 2.71 points.

**Table 3.** Pre- and post-program self-assessed scores for individual RWE research competency areas.

Competency category	Domains	Competency area	*N*	Pre-training score mean (SD)	Post-training score mean (SD)	Change (95% CI)	*P* value	Cohen’s *d*
Foundation knowledge & study design	1. Scientific concepts & research design	Identify research questions for maternal health EHR studies	17	4.88	7.29	2.41 (1.47, 3.36)	<.001***	1.31
		Understand OHDSI study designs	17	2.76	6.71	3.94 (2.68, 5.21)	<.001***	1.6
		Design cohorts using ATLAS	17	2.12	5.82	3.71 (2.41, 5.00)	<.001***	1.47
	2. Ethics & governance	Human subject protection principles	17	5.41	7.82	2.41 (1.34, 3.49)	<.001***	1.15
		Ethics in maternal health research	17	7.76	8.71	0.94 (0.12, 1.76)	.027*	0.59
		Data governance & regulatory requirements	17	4.47	7	2.53 (1.62, 3.44)	<.001***	1.43
	3. Protocol development	Formulate research questions & hypotheses	17	5.82	8.12	2.29 (1.21, 3.38)	<.001***	1.09
		Define inclusion & exclusion criteria	17	5.65	7.94	2.29 (1.11, 3.48)	<.001***	0.99
		Define computable phenotypes	17	4.18	6.88	2.71 (1.70, 3.71)	<.001***	1.38
		Select appropriate data elements	17	6.12	7.82	1.71 (0.78, 2.63)	.001**	0.95
		Develop statistical analysis plans	17	6.71	7.94	1.24 (0.41, 2.06)	.006**	0.77
		Understand protocol components	17	7.29	8.59	1.29 (0.24, 2.35)	.019*	0.63
Operational & technical skills	4. Study operations	Manage multi-institutional study execution	17	2.18	4.94	2.76 (1.83, 3.70)	<.001***	1.52
		Understand OHDSI network team roles	17	3.47	6.18	2.71 (1.45, 3.96)	<.001***	1.11
		Use open-source environments for collaboration research	17	2.82	4	1.18 (0.72, 1.63)	<.001***	1.33
	5. Site management	Coordinate timelines across sites	17	2.82	5.06	2.24 (1.41, 3.06)	<.001***	1.4
		Manage communications with data partners	17	3.18	5.65	2.47 (1.47, 3.47)	<.001***	1.27
		Track study progress across sites	17	2.88	4.94	2.06 (1.12, 2.99)	<.001***	1.13
	6. Informatics & data analysis	Understand OHDSI data flow process	15	2.33	5.8	3.47 (2.32, 4.61)	<.001***	1.68
		Use standardized vocabularies & concept sets	16	1.81	6	4.19 (2.86, 5.51)	<.001***	1.69
		Understand OMOP CDM structure	16	2.19	7.19	5.00 (3.69, 6.31)	<.001***	2.04
		Generating cohorts with OHDSI tool (ATLAS)	16	1.62	6	4.38 (3.21, 5.54)	<.001***	2
		Performing characterization studies	16	1.56	4.62	3.06 (2.06, 4.06)	<.001***	1.63
		Conducting population-level estimation studies	16	1.88	4.38	2.50 (1.57, 3.43)	<.001***	1.43
		Statistical analysis for large-scale RWE studies	16	5.06	6.25	1.19 (0.51, 1.87)	.002**	0.93
Leadership & communication	7. Leadership & professionalism	Take initiative in research planning	16	2	5.56	3.56 (2.48, 4.65)	<.001***	1.75
		Apply OHDSI guidelines & conventions	16	2.19	5.94	3.75 (2.85, 4.65)	<.001***	2.21
	8. Communications & teamwork	Understand network communication protocols	16	2.62	6	3.38 (2.13, 4.62)	<.001***	1.45
		Communicate findings to non-technical audiences	16	6.5	7.94	1.44 (0.37, 2.50)	.012*	0.72

Abbreviations: *N*, number of respondents; RWE, real-world evidence.

Scores based on 0-10 scale where 0 = little knowledge and 10 = high proficiency; Paired *t*-tests used; Cohen’s *d* shows effect size;

*** *P* < .001,

** *P* < .01,

* *P* < .05.

Operational and technical skills showed similar patterns. Understanding roles and responsibilities within OHDSI network study teams and stakeholder engagement increased by 2.71 points. Managing communications and coordination with data partners increased by 2.47 points. The largest change across all competency areas was observed in understanding the OMOP CDM structure and standardized vocabularies for reproducible RWE research across institutions, with a mean of 5.00 points.

Leadership and communication skills also showed notable changes, with a 3.75-point increase in applying OHDSI guidelines for OMOP CDM-based RWE study design and a 3.38-point increase in understanding relationships and communication protocols among OHDSI network collaborators.

### Competency by research experience

**Figure 2.**
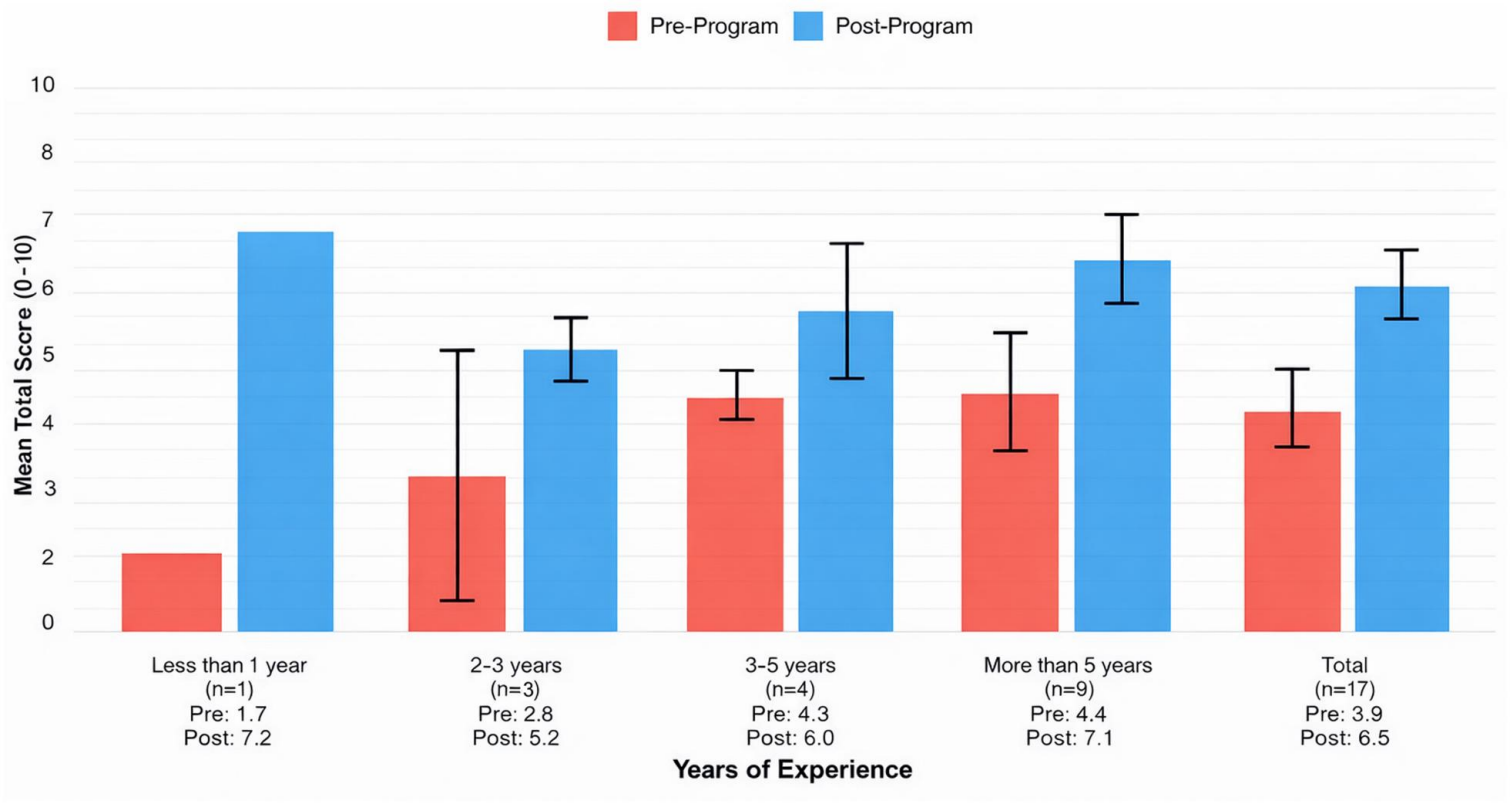
Comparison of pre vs post-training score across years of experience. Pre- and post-training assessment scores stratified by participants’ years of professional experience, demonstrating changes in performance following the training intervention.

When examining competency changes by years of clinical research experience, participants across all experience levels showed increases following the program (Figure 2). Overall mean competency scores increased from 3.9 at baseline to 6.5 post-training. Among experience groups, participants with 2-3 years of experience increased by an average of 2.4 points, those with 3-5 years by 1.7 points, and those with more than 5 years by 2.7 points. While all groups demonstrated gains, participants with more than 5 years of experience showed the largest mean change, followed by those with 2-3 years of experience.

### Competency by professional role

**Figure 3.**
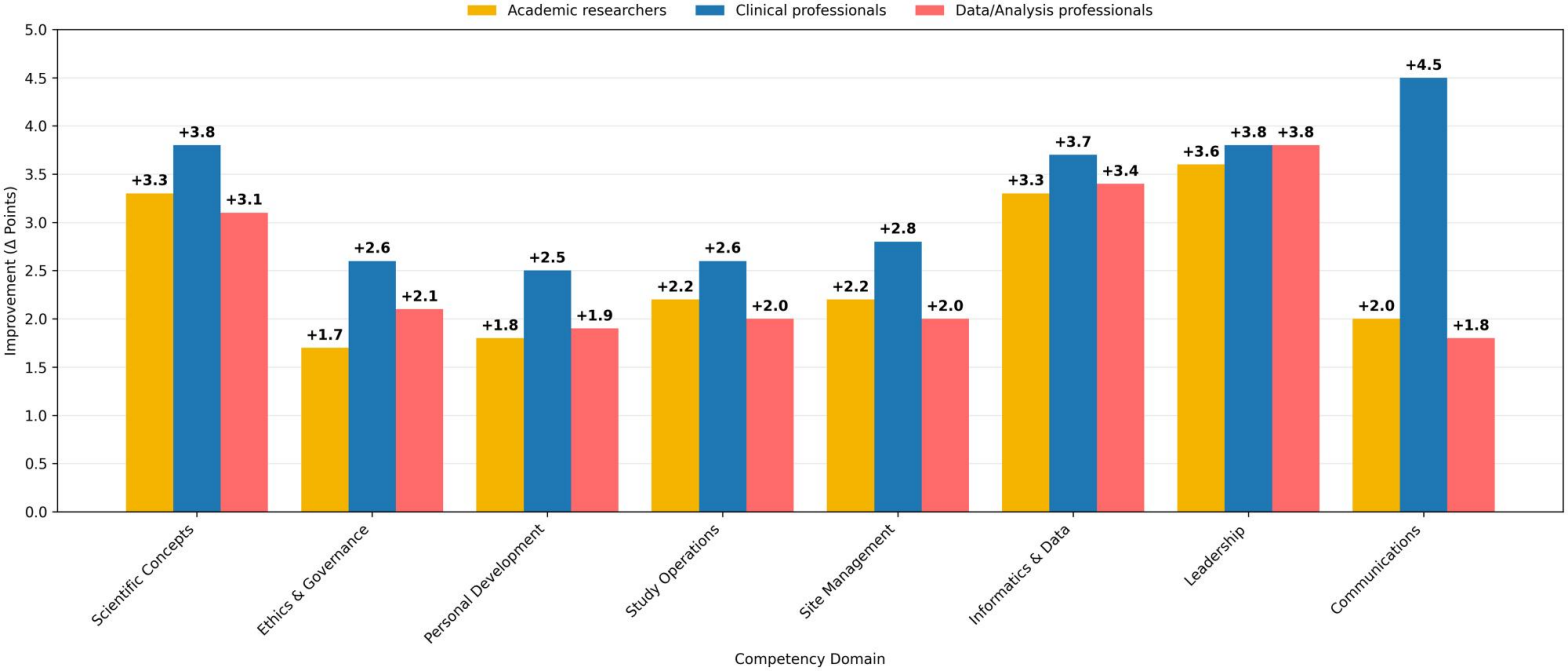
Comparison of pre and post-training scores by professional roles: mean improvement across domains. Mean pre- and post-training scores across evaluated domains according to participants’ professional roles, illustrating overall improvement after training.

Self-assessed competency changes varied by professional role (Figure 3). Clinical professionals showed the largest increase of 4.5 points in Communications and Teamwork (Domain 8), along with notable changes in Scientific Concepts and Research Design (3.8 points) and Leadership and Professionalism (3.8 points). Academic researchers showed increases ranging from 1.7 to 3.6 points across domains, with the largest gains in Leadership and Professionalism (Domain 7). Similarly, data/analysis professionals showed their largest increase of 3.8 points in Domain 7.

## Discussion

This study assessed competencies related to conducting large-scale RWE studies using standardized healthcare data by developing a 29-item competency instrument and applying it to measure changes in trainees’ self-reported skills. Respondents from academia, healthcare systems, and data/analytics roles reported changes in their competencies following the OHDSI maternal health fellowship program, regardless of their years of experience or professional background. Across the 8 competency domains, participants showed an average increase of approximately 4 points, with the larger changes in leadership and professionalism. These findings illustrate that the competency assessment instrument can serve as a practical tool for evaluating RWE researchers and identifying areas where additional training and workforce development may be needed.

The largest changes were observed in the domain of Leadership and Professionalism, followed by Informatics and Data Analysis and Scientific Concepts and Research Design. OHDSI-based studies typically involve multidisciplinary teams, including epidemiologists, data scientists, statisticians, clinicians, informaticians, and project managers, and are often conducted across multiple institutions and time zones.^12^^,^^34^ Within such settings, researchers are required to coordinate distributed workflows, maintain communication across roles, and align diverse perspectives toward shared study objectives. In addition, RWE research requires discussion and consensus among study teams during the development of study protocols, the development of analytic strategies, and the interpretation of results, and such collaborative and consensus-driven research activities call for leadership-related competencies, including coordination, communication, and shared decision-making.^35^ The observed increases in Leadership and Professionalism may be related to increased exposure to these collaborative research environments and may reflect a broader understanding of the roles and responsibilities involved in conducting large-scale RWE studies.

Another notable finding was the increase in the domain of Informatics and Data Analysis, indicating that participants received training in technical competencies that are lacking in traditional clinical research education. In this study, the fellowship provided education on understanding the structure of the OMOP CDM, using standardized vocabularies, navigating OHDSI data flows, and implementing open-source analytic tools to build applied informatics and data analysis skills. The hands-on, practice-oriented design of the fellowship, including the use of OHDSI open-source tools such as Analytics plaTform for Large-scale AnalysiS (ATLAS) and Athena (OHDSI Standardized Vocabularies), may have further supported participants’ ability to work with complex data models and standardized analytic pipelines. Increases were also observed in Scientific Concepts and Research Design, suggesting a greater understanding of OHDSI-based RWE study design. These gains may be related to curriculum components that introduced a standardized, open-source framework for conducting RWE research across multiple analytic objectives, including descriptive analyses, population-level estimation, and patient-level prediction.

Domain 2 showed smaller increases than other domains, likely due to participants’ higher baseline knowledge and its overlap with standard clinical trial training. However, the lower end-of-fellowship scores suggest that additional or alternative training in human subjects protection, data governance, and regulatory requirements may be needed. These competencies differ from technical or analytic skills in that they are highly context-dependent and shaped by institutional policies, legal frameworks, and local regulatory environments. In RWE research using EHR data, ethical review processes, data use agreements, and governance requirements vary across institutions and countries, limiting the extent to which standardized training alone can translate into immediate competency gains.^36^^,^^37^ In addition, although RWE studies using de-identified or limited datasets may in some cases qualify for exemption or expedited review, complex and variable IRB processes may limit opportunities to develop related competenceis during short training programs.^38^ These factors highlight the need for longitudinal, institutionally embedded training approaches to support competency development in research ethics and data governance.

Our study has several limitations. First, the small sample size of 17 participants limits the generalizability of the results. Second, competencies were assessed using self-reported measures, which are subject to social desirability bias and variability in how participants interpret and apply rating scales, potentially influencing the accuracy of the reported changes. In addition, with a retrospective pre-post design, observed increases may reflect exposure to training content or familiarity bias rather than deeper or practically applied understanding, and learning gains may be overestimated. Third, although the survey instrument was developed based on content validity, additional evaluation of the validity of the assessment instrument, including construct and criterion validity, is warranted. Lastly, as assessments were collected at a single time point, the analysis does not capture how competencies evolved over the course of the program. Future work could incorporate assessments at multiple time points and use scenario or case-based questions to better assess deeper understanding and the ability to apply knowledge.

## Conclusion

This study developed and evaluated a competency assessment framework for RWE research, providing a standardized approach for assessing workforce training to support RWE studies. Analysis of pre- and post-training surveys showed increases in self-reported competency across multiple domains, with larger increases in Leadership and Professionalism and in Informatics and Data Analysis than in other domains. These findings indicate that a competency-based assessment approach can help describe patterns of skill development associated with RWE research training and clarify areas where additional training may be needed. This framework may be used to monitor competencies in RWE research training and to inform the development of key competency areas for RWE researchers.

## Supplementary Material

ooag128_Supplementary_Data

## Data Availability

The survey data used in this study may be shared upon request while protecting participant confidentiality.
