# Supplementary material for: Assessment of real-world evidence research competencies in federated research networks: a maternal health fellowship program evaluation
Source: JAMIA Open. 2026 Jul 14;9(4):ooag128. doi: 10.1093/jamiaopen/ooag128 (PMC13368817; doi:10.1093/jamiaopen/ooag128)
Supplement: ooag128_Supplementary_Data [file ooag128_supplementary_data.docx]

| **Appendix 1. Detailed Descriptions of Competencies Mapped from JTF to RWE Research Competency Domains** | | | |
| --- | --- | --- | --- |
| **JTF Competency Domains** | **JTF Competency Description** | **RWE Competency Domain** | **RWE Competency Description** |
| 1. Scientific concepts and research design | Identify clinically important questions that are potentially testable clinical research hypotheses, through review of the professional literature | 1. Scientific concepts and research design | Identify clinically important research questions suitable for RWE studies through systematic review of professional literature and assessment of real-world data capabilities. |
|  | Explain the elements (statistical, epidemiological and operational) of clinical translational study design |  | Explain the elements (statistical, and epidemiological) of RWE study designs, including characterization studies, and population-level estimation. |
|  | Critically analyze study results with an understanding of therapeutic and comparative effectiveness |  | Design and implement target and comparator cohorts using standardized tools and critically analyze RWE study results with understanding of real-world data effectiveness and population health implications. |
| 2. Ethical and participant safety considerations | Compare the requirements for human subject protection and privacy under different national and international regulations and ensure their implementation throughout all phases of a clinical study | 2. RWE research ethics and governance | Understand human subject protection principles in multi-institutional RWE studies using standardized EHR databases and ensure their implementation throughout all phases of RWE research. |
|  | Describe the ethical issues involved when dealing with vulnerable populations and the need for additional safeguards |  | Compare the requirements for data governance and regulatory compliance under different institutional and national regulations and ensure their implementation throughout all phases of multi-institutional RWE research. |
| 3. Investigational products development and regulation | Describe the roles and responsibilities of the various institutions participating in the medicine development process | 3. RWE research protocol development | Formulate clear research questions and testable hypotheses for cross-institutional RWE research that can be effectively implemented across diverse healthcare systems. |
|  |  |  | Define study populations through appropriate inclusion/exclusion criteria and develop computable phenotypes using standardized healthcare data elements. |
|  |  |  | Develop an analysis plan and RWE research protocol, including the selection of appropriate statistical methods and the integration of necessary protocol components. |
| 4. Clinical study operations (Good clinical practice) | Evaluate the conduct and management of clinical trials within the context of a clinical development plan | 4. RWE study operations | Evaluate the conduct and management of multi-institutional RWE studies within the context of OHDSI network research frameworks |
|  | Describe the roles and responsibilities of the clinical investigation team as defined by Good Clinical Practice Guidelines |  | Describe the roles and responsibilities of the RWE research investigation team as defined by OHDSI collaborative research guidelines. |
|  | Describe the role and process of monitoring for the study |  | Describe the role and process of collaborative platforms and version control systems for ensuring reproducibility and transparency in RWE studies. |
| 5. Study and site management | Develop and manage the financial, timeline and cross-disciplinary personnel resources necessary to conduct a clinical or translational research study | 5. Study and Site Management | Develop and manage the timeline and cross-disciplinary personnel resources necessary to conduct multi-institutional RWE research studies. |
|  | Apply management concepts and effective training methods to manage risk and improve quality in the conduct of a clinical research study |  | Apply management concepts and effective communication methods to manage risk and improve quality in the conduct of RWE research studies. |
|  | Utilize elements of project management related of study site organization to manage patient recruitment, complete procedures and track progress |  | Utilize elements of project management related to study site organization to manage cohort implementation, complete analytical procedures and track progress. |
| 6. Data management and informatics | Describe the role that biostatistics and informatics serve in biomedical and public health research | 6. Data analysis and informatics | Describe the typical flow of data throughout RWE studies and the role of common data models in enabling scalable research. |
|  | Describe the typical flow of data throughout a clinical trial |  | Summarize the process of standardized data processing and the importance of open-source tools in data harmonization and analysis for RWE research. |
|  | Summarize the process of electronic data capture (EDC) and the importance of information technology in data collection, capture and management |  | Describe appropriate statistical methods for large-scale RWE analyses |
|  | Describe the significance of data quality assurance systems and how SOPs are used to guide these processes |  | Apply standardized analytical frameworks and ensure reproducibility standards across multi-institutional RWE research networks. |
| 7. Leadership and professionalism | Describe the principles and practices of leadership, management and mentorship, and apply them within the working environment | 7. Leadership and professionalism | Describe the principles and practices of leadership, management and mentorship for RWE research teams, and apply them within healthcare and research settings. |
|  | Identify and apply the professional guidelines and codes of ethics that apply to the conduct of clinical research |  | Identify and apply the professional guidelines and codes of ethics that apply to the conduct of RWE research within collaborative networks. |
| 8. Communications and Teamwork | Discuss the relationship and appropriate communication between sponsor, CRO and clinical research site | 8. Communication and Teamwork | Discuss the relationship and appropriate communication between study leads, data partner institutions, and OHDSI network collaborators in RWE research. |
|  | Effectively communicate the content and relevance of clinical research findings to colleagues, advocacy groups and the non-scientist community |  | Effectively communicate the content and relevance of clinical research findings to colleagues, clinicians, advocacy groups and the non-scientist community |
| Note: JTF = Joint Task Force for Clinical Trial Competency; RWE = Real-World Evidence; RWD = Real-World Data; OMOP = Observational Medical Outcomes Partnerships; OHDSI = Observational Health Data Sciences and Informatics; EHR = Electronic Health Record; GCP = Good Clinical Practice; CDM = Common Data Model | | | |

**Appendix 2. Real-World Evidence (RWE) Research Competency Survey Instrument**

OHDSI Maternal Health Fellowship End of Course Survey

Welcome

Thank you in advance for completing this survey. This survey aims to provide feedback on your experience in the OHDSI Maternal Health Data Science Fellowship. The program is designed to train Maternal Health researchers in using standardized EHR data to conduct RWE research across institutions. Your feedback is vital to us as this is the first year we offered the program and our hope it to tailor what we learn for the next cohort of students.

Instructions: For each competency area below, please rate your ability level BEFORE starting the fellowship and NOW after completing the fellowship using the following scale:

Rating Scale:

- 1-2: Beginner (Little to no knowledge)
- 3-4: Limited knowledge (Basic concepts only)
- 5-6: Intermediate (Some practical application)
- 7-8: Advanced (Independent performance)
- 9-10: Expert (Can teach and guide others)

Completing this survey or questionnaire will serve as consent to be in this research study. This survey should take about 15 minutes to complete.

**Background**

1. What is the highest degree you earned before joining this fellowship?

**☐**Bachelor’s degree

**☐**Master’s degree

**☐**Doctoral degree

**☐**Other: ___________

1. How many years of clinical research experience do you have?

☐ Less than 1 year

☐ 1-2 years

☐ 2-3 years

☐ 3-5 years

☐ More than 5 years

1. What’s your current job title or role?

**☐**Clinician

**☐**Data scientist

**☐**Informatician

**☐**Biostatistician

**☐**Other: __________

**1. Scientific Concepts and Research Design**

Q1. How well can you Identify and evaluate clinically important research questions suitable for RWE studies in maternal health using standardized EHR data?

|  | 1 | 2 | 3 | 4 | 5 | 6 | 7 | 8 | 9 | 10 |
| --- | --- | --- | --- | --- | --- | --- | --- | --- | --- | --- |
| BEFORE the program | ○ | ○ | ○ | ○ | ○ | ○ | ○ | ○ | ○ | ○ |
| NOW after the program | ○ | ○ | ○ | ○ | ○ | ○ | ○ | ○ | ○ | ○ |

Q2 To what extent do you understand OHDSI study designs for cross-institutional RWE research (characterization, population-level estimation, patient-level prediction)?

|  | 1 | 2 | 3 | 4 | 5 | 6 | 7 | 8 | 9 | 10 |
| --- | --- | --- | --- | --- | --- | --- | --- | --- | --- | --- |
| BEFORE the program | ○ | ○ | ○ | ○ | ○ | ○ | ○ | ○ | ○ | ○ |
| NOW after the program | ○ | ○ | ○ | ○ | ○ | ○ | ○ | ○ | ○ | ○ |

Q3 How proficient are you in designing study population and outcome cohorts using ATLAS?

|  | 1 | 2 | 3 | 4 | 5 | 6 | 7 | 8 | 9 | 10 |
| --- | --- | --- | --- | --- | --- | --- | --- | --- | --- | --- |
| BEFORE the program | ○ | ○ | ○ | ○ | ○ | ○ | ○ | ○ | ○ | ○ |
| NOW after the program | ○ | ○ | ○ | ○ | ○ | ○ | ○ | ○ | ○ | ○ |

**2. RWE Research Ethnics and Governance**

Q4 How well do you understand human subject projection principles in multi-institutional RWE studies using standardized EHR databases? (eg., Informed consent (waived for de-identified data), privacy & confidentiality, IRB review)

|  | 1 | 2 | 3 | 4 | 5 | 6 | 7 | 8 | 9 | 10 |
| --- | --- | --- | --- | --- | --- | --- | --- | --- | --- | --- |
| BEFORE the program | ○ | ○ | ○ | ○ | ○ | ○ | ○ | ○ | ○ | ○ |
| NOW after the program | ○ | ○ | ○ | ○ | ○ | ○ | ○ | ○ | ○ | ○ |

Q5 To what extent do you understand the ethical considerations specific to maternal health research with vulnerable populations?

|  | 1 | 2 | 3 | 4 | 5 | 6 | 7 | 8 | 9 | 10 |
| --- | --- | --- | --- | --- | --- | --- | --- | --- | --- | --- |
| BEFORE the program | ○ | ○ | ○ | ○ | ○ | ○ | ○ | ○ | ○ | ○ |
| NOW after the program | ○ | ○ | ○ | ○ | ○ | ○ | ○ | ○ | ○ | ○ |

Q6 How well do you understand the data governance frameworks and regulatory requirements for multi-institutional RWE research using EHR? (eg., Data Access and Use Policies, Data Ownership and Stewardship, Data Sharing Agreements, Privacy and Confidentiality Controls (HIPAA Compliance), IRB/Ethics)

|  | 1 | 2 | 3 | 4 | 5 | 6 | 7 | 8 | 9 | 10 |
| --- | --- | --- | --- | --- | --- | --- | --- | --- | --- | --- |
| BEFORE the program | ○ | ○ | ○ | ○ | ○ | ○ | ○ | ○ | ○ | ○ |
| NOW after the program | ○ | ○ | ○ | ○ | ○ | ○ | ○ | ○ | ○ | ○ |

**3. RWE Research Protocol Development**

Q7 How confident are you in formulating clear research questions and testable hypotheses for cross-institutional RWE research? (eg., Writing a clear, focused research question,  Creating testable hypotheses,  Designing studies that work across institutions)

|  | 1 | 2 | 3 | 4 | 5 | 6 | 7 | 8 | 9 | 10 |
| --- | --- | --- | --- | --- | --- | --- | --- | --- | --- | --- |
| BEFORE the program | ○ | ○ | ○ | ○ | ○ | ○ | ○ | ○ | ○ | ○ |
| NOW after the program | ○ | ○ | ○ | ○ | ○ | ○ | ○ | ○ | ○ | ○ |

Q8 How confident are you in identifying appropriate inclusion/exclusion criteria using standardized EHR data across institutions?

|  | 1 | 2 | 3 | 4 | 5 | 6 | 7 | 8 | 9 | 10 |
| --- | --- | --- | --- | --- | --- | --- | --- | --- | --- | --- |
| BEFORE the program | ○ | ○ | ○ | ○ | ○ | ○ | ○ | ○ | ○ | ○ |
| NOW after the program | ○ | ○ | ○ | ○ | ○ | ○ | ○ | ○ | ○ | ○ |

Q9 How well can you define computable phenotypes (e.g., outcomes or conditions) for use in RWE research using standardized healthcare data?

|  | 1 | 2 | 3 | 4 | 5 | 6 | 7 | 8 | 9 | 10 |
| --- | --- | --- | --- | --- | --- | --- | --- | --- | --- | --- |
| BEFORE the program | ○ | ○ | ○ | ○ | ○ | ○ | ○ | ○ | ○ | ○ |
| NOW after the program | ○ | ○ | ○ | ○ | ○ | ○ | ○ | ○ | ○ | ○ |

Q10 How well can you identify and select appropriate data elements (e.g., exposures, outcomes, covariates) to support your RWE study design?

|  | 1 | 2 | 3 | 4 | 5 | 6 | 7 | 8 | 9 | 10 |
| --- | --- | --- | --- | --- | --- | --- | --- | --- | --- | --- |
| BEFORE the program | ○ | ○ | ○ | ○ | ○ | ○ | ○ | ○ | ○ | ○ |
| NOW after the program | ○ | ○ | ○ | ○ | ○ | ○ | ○ | ○ | ○ | ○ |

Q11 How well can you develop an analysis plan for an RWE study, including the selection of statistical methods (e.g., Logistic regression, survival analysis, Propensity score matching, etc) and strategies to address bias and confounding (e.g., sensitivity analyses)?

|  | 1 | 2 | 3 | 4 | 5 | 6 | 7 | 8 | 9 | 10 |
| --- | --- | --- | --- | --- | --- | --- | --- | --- | --- | --- |
| BEFORE the program | ○ | ○ | ○ | ○ | ○ | ○ | ○ | ○ | ○ | ○ |
| NOW after the program | ○ | ○ | ○ | ○ | ○ | ○ | ○ | ○ | ○ | ○ |

Q12 How well do you understand the components of an RWEresearch protocol (eg., background, objectives, study design, population, data sources, outcomes, analytic plan, bias mitigation)?

|  | 1 | 2 | 3 | 4 | 5 | 6 | 7 | 8 | 9 | 10 |
| --- | --- | --- | --- | --- | --- | --- | --- | --- | --- | --- |
| BEFORE the program | ○ | ○ | ○ | ○ | ○ | ○ | ○ | ○ | ○ | ○ |
| NOW after the program | ○ | ○ | ○ | ○ | ○ | ○ | ○ | ○ | ○ | ○ |

**4. RWE Study Operations**

Q13 How confident are you in managing the execution of multi-institutional OHDSI network studies, including coordinating timelines, deliverables, communication, and governance across data partner sites?

|  | 1 | 2 | 3 | 4 | 5 | 6 | 7 | 8 | 9 | 10 |
| --- | --- | --- | --- | --- | --- | --- | --- | --- | --- | --- |
| BEFORE the program | ○ | ○ | ○ | ○ | ○ | ○ | ○ | ○ | ○ | ○ |
| NOW after the program | ○ | ○ | ○ | ○ | ○ | ○ | ○ | ○ | ○ | ○ |

Q14 To what extent can you understand the roles and responsibilities within an OHDSI network study team (e.g., coordinating investigator, analytic lead, data partner lead, clinical lead, statistician)?

|  | 1 | 2 | 3 | 4 | 5 | 6 | 7 | 8 | 9 | 10 |
| --- | --- | --- | --- | --- | --- | --- | --- | --- | --- | --- |
| BEFORE the program | ○ | ○ | ○ | ○ | ○ | ○ | ○ | ○ | ○ | ○ |
| NOW after the program | ○ | ○ | ○ | ○ | ○ | ○ | ○ | ○ | ○ | ○ |

Q15 To what extent are you able to use GitHub to support collaborative cross-institutional RWE research (e.g., code versioning, issue tracking, protocol sharing, ensuring reproducibility)?

|  | 1 | 2 | 3 | 4 | 5 | 6 | 7 | 8 | 9 | 10 |
| --- | --- | --- | --- | --- | --- | --- | --- | --- | --- | --- |
| BEFORE the program | ○ | ○ | ○ | ○ | ○ | ○ | ○ | ○ | ○ | ○ |
| NOW after the program | ○ | ○ | ○ | ○ | ○ | ○ | ○ | ○ | ○ | ○ |

**5. Study and Site Management**

Q16 How well can you coordinate timelines and deliverables across sites in a multi-institutional OHDSI study?

|  | 1 | 2 | 3 | 4 | 5 | 6 | 7 | 8 | 9 | 10 |
| --- | --- | --- | --- | --- | --- | --- | --- | --- | --- | --- |
| BEFORE the program | ○ | ○ | ○ | ○ | ○ | ○ | ○ | ○ | ○ | ○ |
| NOW after the program | ○ | ○ | ○ | ○ | ○ | ○ | ○ | ○ | ○ | ○ |

Q17 How comfortable are you managing communication and coordination with external data partners to ensure consistent local execution of OHDSI study protocols?

|  | 1 | 2 | 3 | 4 | 5 | 6 | 7 | 8 | 9 | 10 |
| --- | --- | --- | --- | --- | --- | --- | --- | --- | --- | --- |
| BEFORE the program | ○ | ○ | ○ | ○ | ○ | ○ | ○ | ○ | ○ | ○ |
| NOW after the program | ○ | ○ | ○ | ○ | ○ | ○ | ○ | ○ | ○ | ○ |

Q18 How well can you track the progress of RWE study activities (e.g., cohort implementation, result generation) across study sites?

|  | 1 | 2 | 3 | 4 | 5 | 6 | 7 | 8 | 9 | 10 |
| --- | --- | --- | --- | --- | --- | --- | --- | --- | --- | --- |
| BEFORE the program | ○ | ○ | ○ | ○ | ○ | ○ | ○ | ○ | ○ | ○ |
| NOW after the program | ○ | ○ | ○ | ○ | ○ | ○ | ○ | ○ | ○ | ○ |

**6. Informatics and Data Analysis**

Q19 How well do you understand the flow of standardized data during an OHDSI RWE study from cohort definition, covariate selection, and characterization to outcome analyses?

|  | 1 | 2 | 3 | 4 | 5 | 6 | 7 | 8 | 9 | 10 |
| --- | --- | --- | --- | --- | --- | --- | --- | --- | --- | --- |
| BEFORE the program | ○ | ○ | ○ | ○ | ○ | ○ | ○ | ○ | ○ | ○ |
| NOW after the program | ○ | ○ | ○ | ○ | ○ | ○ | ○ | ○ | ○ | ○ |

Q20 How well can you use standardized vocabularies (e.g., SNOMED, LOINC, RxNorm) and concept sets in OHDSI tools (e.g., ATLAS, ATHENA) to identify exposures, outcomes, and covariates?

|  | 1 | 2 | 3 | 4 | 5 | 6 | 7 | 8 | 9 | 10 |
| --- | --- | --- | --- | --- | --- | --- | --- | --- | --- | --- |
| BEFORE the program | ○ | ○ | ○ | ○ | ○ | ○ | ○ | ○ | ○ | ○ |
| NOW after the program | ○ | ○ | ○ | ○ | ○ | ○ | ○ | ○ | ○ | ○ |

Q21 How well do you understand the role of the OMOP CDM structure and standard vocabularies in supporting scalable, reproducible observational research across institutions?

|  | 1 | 2 | 3 | 4 | 5 | 6 | 7 | 8 | 9 | 10 |
| --- | --- | --- | --- | --- | --- | --- | --- | --- | --- | --- |
| BEFORE the program | ○ | ○ | ○ | ○ | ○ | ○ | ○ | ○ | ○ | ○ |
| NOW after the program | ○ | ○ | ○ | ○ | ○ | ○ | ○ | ○ | ○ | ○ |

Q22 How comfortable are you using OHDSI tools (e.g., ATLAS) to define cohorts, develop phenotypes, and implement observational studies?

|  | 1 | 2 | 3 | 4 | 5 | 6 | 7 | 8 | 9 | 10 |
| --- | --- | --- | --- | --- | --- | --- | --- | --- | --- | --- |
| BEFORE the program | ○ | ○ | ○ | ○ | ○ | ○ | ○ | ○ | ○ | ○ |
| NOW after the program | ○ | ○ | ○ | ○ | ○ | ○ | ○ | ○ | ○ | ○ |

Q23 How well can you conduct characterization tasks such as feature analysis, cohort pathway analysis, and incidence rate estimation?

|  | 1 | 2 | 3 | 4 | 5 | 6 | 7 | 8 | 9 | 10 |
| --- | --- | --- | --- | --- | --- | --- | --- | --- | --- | --- |
| BEFORE the program | ○ | ○ | ○ | ○ | ○ | ○ | ○ | ○ | ○ | ○ |
| NOW after the program | ○ | ○ | ○ | ○ | ○ | ○ | ○ | ○ | ○ | ○ |

 Q24 How confident are you conduct population-level estimation studies using target-comparator designs, negative controls, and effect estimation techniques?

|  | 1 | 2 | 3 | 4 | 5 | 6 | 7 | 8 | 9 | 10 |
| --- | --- | --- | --- | --- | --- | --- | --- | --- | --- | --- |
| BEFORE the program | ○ | ○ | ○ | ○ | ○ | ○ | ○ | ○ | ○ | ○ |
| NOW after the program | ○ | ○ | ○ | ○ | ○ | ○ | ○ | ○ | ○ | ○ |

Q25 How would you describe your understanding of statistical methods for large-scale RWE analyses (e.g., propensity scoring, stratifications, etc.)?

|  | 1 | 2 | 3 | 4 | 5 | 6 | 7 | 8 | 9 | 10 |
| --- | --- | --- | --- | --- | --- | --- | --- | --- | --- | --- |
| BEFORE the program | ○ | ○ | ○ | ○ | ○ | ○ | ○ | ○ | ○ | ○ |
| NOW after the program | ○ | ○ | ○ | ○ | ○ | ○ | ○ | ○ | ○ | ○ |

**7. Leadership and professionalism**

Q26 Are you confident in taking initiative in planning and organizing RWE research using OMOP CDM (e.g., defining objectives, selecting data elements, outlining study plans)?

|  | 1 | 2 | 3 | 4 | 5 | 6 | 7 | 8 | 9 | 10 |
| --- | --- | --- | --- | --- | --- | --- | --- | --- | --- | --- |
| BEFORE the program | ○ | ○ | ○ | ○ | ○ | ○ | ○ | ○ | ○ | ○ |
| NOW after the program | ○ | ○ | ○ | ○ | ○ | ○ | ○ | ○ | ○ | ○ |

Q27 How well can you apply OHDSI guidelines or conventions (e.g., transparency, reproducibility, standardization, open science and collaboration) when designing an RWE study using the OMOP CDM?

|  | 1 | 2 | 3 | 4 | 5 | 6 | 7 | 8 | 9 | 10 |
| --- | --- | --- | --- | --- | --- | --- | --- | --- | --- | --- |
| BEFORE the program | ○ | ○ | ○ | ○ | ○ | ○ | ○ | ○ | ○ | ○ |
| NOW after the program | ○ | ○ | ○ | ○ | ○ | ○ | ○ | ○ | ○ | ○ |

**8. Communication and Teamwork**

Q28 How well do you understand the relationships and communication protocols between study leads, data partner institutions, and OHDSI network collaborators in RWE studies?

|  | 1 | 2 | 3 | 4 | 5 | 6 | 7 | 8 | 9 | 10 |
| --- | --- | --- | --- | --- | --- | --- | --- | --- | --- | --- |
| BEFORE the program | ○ | ○ | ○ | ○ | ○ | ○ | ○ | ○ | ○ | ○ |
| NOW after the program | ○ | ○ | ○ | ○ | ○ | ○ | ○ | ○ | ○ | ○ |

Q29 How well can you communicate RWE research findings and their clinical implications to non-technical audiences, including clinicians, patient advocacy groups, and interdisciplinary colleagues?

|  | 1 | 2 | 3 | 4 | 5 | 6 | 7 | 8 | 9 | 10 |
| --- | --- | --- | --- | --- | --- | --- | --- | --- | --- | --- |
| BEFORE the program | ○ | ○ | ○ | ○ | ○ | ○ | ○ | ○ | ○ | ○ |
| NOW after the program | ○ | ○ | ○ | ○ | ○ | ○ | ○ | ○ | ○ | ○ |

**Program Evaluation Questions**

1.  How well did the skills and knowledge gained align with your career development needs?

☐ Very poorly

☐ Poorly

☐ Natural

☐ Well

☐ Very well

2. How effective was the fellowship in helping you build relationships with potential collaborators?

☐ Not effective

☐ Slightly effective

☐ Moderately effective

☐ Very effective

☐ Extremely effective

3. How would you describe the advisory relationships you developed through the Maternal Health Fellowship in terms of supporting your career goals?

☐ No advisory relationships developed

☐ Minimal support for my career goals

☐ Some support, but limited

☐ Helpful and somewhat supportive

☐ Very helpful and strongly supportive

4. What improvements would you suggest for the next cohort of the OHDSI Maternal Health Fellowship? (e.g., Curriculum, delivery, resources, more hands-on session using ATLAS)

___________________________________________________________________

5. Are there any additional topics or tools related to standardized EHR data analysis you would like to see included in the curriculum?

___________________________________________________________________


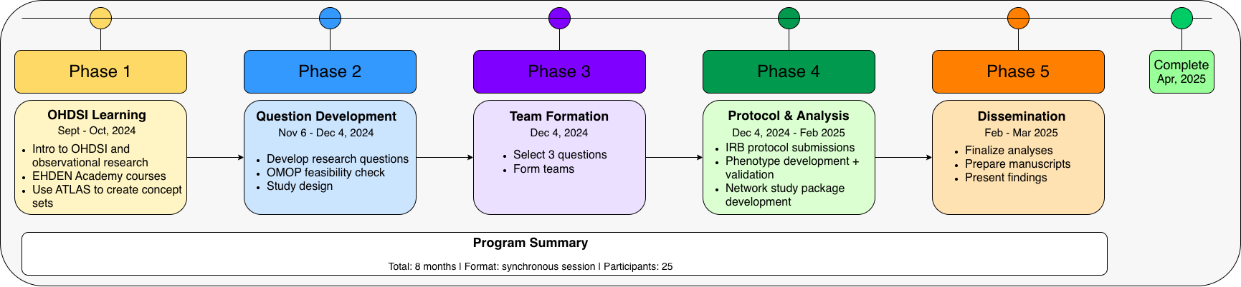


**Appendix 3. OHDSI Maternal Health Fellowship Training Program Structure**
